# Erythropoietin rs1617640 G allele associates with an attenuated rise of serum erythropoietin and a marked decline of hemoglobin in hepatitis C patients undergoing antiviral therapy

**DOI:** 10.1186/1471-2334-14-503

**Published:** 2014-09-17

**Authors:** Ahmad Amanzada, Armin D Goralczyk, Lars Reinhardt, Federico Moriconi, Silke Cameron, Sabine Mihm

**Affiliations:** Department of Gastroenterology and Endocrinology, University Medical Center Goettingen, Robert-Koch-Strasse 40, 37075 Goettingen, Germany; Department of Internal Medicine, Clinic of Herzberg and Osterode, Herzberg am Harz, Germany

**Keywords:** Anemia, Chronic hepatitis C virus infection, Ribavirin, *EPO* promoter polymorphism rs1617640, *ITPA* rs1127354

## Abstract

**Background:**

A decline in hemoglobin (Hb) concentration during antiviral therapy in chronic hepatitis C (CHC) is a serious side effect. It may compel to dose reduction or even termination of antiviral treatment. The activation of erythropoietin (EPO) synthesis as a physiological response to anemia and its relation to a genetic variation within the EPO gene has not been evaluated yet.

**Methods:**

Data of 348 CHC patients were reviewed retrospectively. Samples were genotyped for *EPO* rs1617640 and *inosine triphosphatase* (*ITPA*) rs1127354. Serum EPO concentrations were determined before and during therapy. Primary endpoints were set as Hb decline >3 g/dl at weeks 4 and 12.

**Results:**

*EPO* rs1617640 G homozygotes showed a significantly lower rise of serum EPO level over time than T allele carriers (p < 0.001). The cumulative frequency of a significant Hb reduction added up to 40%. Multivariate analysis revealed that besides age, ribavirin starting dose and baseline Hb also *EPO* rs1617640 G homozygosity associates with Hb reduction at week 4 (p = 0.025) and 12 (p = 0.029), while *ITPA* C homozygotes are at risk for Hb decline particularly early during treatment. Furthermore, *EPO* rs1617640 G homozygotes were more frequently in need for blood transfusion, epoetin-α supplementation, or ribavirin dose reduction (p < 0.001).

**Conclusions:**

Our data suggest that *EPO* rs1617640 genotype, the rise of serum EPO concentration as well as *ITPA* rs1127354 genotype are promising parameters to evaluate the Hb decline during antiviral therapy. A rational adjustment of therapy with epoetin-α supplementation might prevent serious adverse events or the need to terminate treatment.

**Electronic supplementary material:**

The online version of this article (doi:10.1186/1471-2334-14-503) contains supplementary material, which is available to authorized users.

## Background

Antiviral combination therapy consisting of pegylated interferon-α and ribavirin (PEG-IFN-α/RBV) for treatment of chronic hepatitis C virus (CHC) infection is highly effective but it is also difficult to tolerate in some patients. In fact, it is associated with significant morbidity and with treatment-limiting adverse events [1]. One important treatment-limiting adverse event is anemia. In various prospective trials dose modification of RBV because of hemoglobin (Hb) reduction were required in 9% up to 22% of patients [2, 3] affecting the overall treatment outcome. Recently, clinical studies assessing efficacy of HCV protease inhibitors in combination with PEG-IFN-α/RBV revealed an even higher rate of anemia ranging between 27%-46% [4, 5]. Moreover, the need to administer erythropoietin (EPO) was also increased about two-fold (up to 46% of boceprevir-treated vs 21% of controls) [5].

IFN-α monotherapy may induce a significant and rapid Hb decrease most probably caused by bone marrow inhibition [6]. RBV, by contrast, contributes to anemia by increasing hemolysis [7]. Several reports have examined serum EPO levels during antiviral treatment and could show an increase up to 4-fold at week 4 in patients treated with PEG-IFN-α_2a_ and RBV while Hb levels are declining [8–12]. In the study by Trivedi et al. [10] the mean EPO serum level increased from 14.5 ± 15.1 at baseline to 58.5 ± 94.1 mIU/ml at week 4 in 43 chronic HCV infected patients treated with antiviral combination therapy. Durante et al. [12] investigated EPO serum concentrations during antiviral combination therapy related to Hb decrease in 18 chronic HCV patients. The mean EPO serum level at the Hb nadir was 55.5 ± 30.5 mIU/ml. Another study could also show that the median EPO serum level increased at week 12 to 41 mIU/ml (range 12–683 mIU/ml) in 145 patients with chronic hepatitis C during PEG-IFN-α and RBV therapy [9, 10]. Of note, a genetic variation within the EPO gene promoter region, rs1617640, was reported to be related to EPO concentration in the vitreous body fluid of non-diabetic patients [13]. In 2010, a genome-wide association study revealed that two functional variants in the *inosine triphosphatase* (*ITPA*) gene causing *ITPA* deficiency protect against RBV-induced hemolytic anemia and the need for RBV dose reduction in patients with HCV genotype 1 infection [14]. Recently, various studies could confirm these findings in CHC genotype 1 to 4 infected patients [15–18]. *ITPA* variants could predict Hb decline during therapy in patients treated with PEG-IFN-α/RBV as well as in patients treated Telaprevir and PEG-IFN-α/RBV [19]. However, the exact mechanism of Hb reduction under combined antiviral therapy in CHC patients is still not fully understood.

This study sought to extend the understanding of Hb decline in CHC patients undergoing antiviral combination therapy. For this purpose, Hb and serum EPO concentrations were monitored before and at week 4, 8 and 12 after onset of antiviral combination therapy and related to *EPO* rs1617640 and *ITPA* rs1127354 genotypes.

## Methods

### Patients and inclusion criteria

Patients were included in this retrospective analysis in which core data and samples were collected before and on treatment. Inclusion criteria for this analysis were HCV-RNA positivity for more than 6 months, treatment with PEG-IFN-α and RBV, age 18 years or older, and compensated liver disease (Child-Pugh score <7). Also blood samples for genotyping and complete data sets for pre- and on-treatment (week 4, 8 and 12) Hb values had to be available. Patients with active hepatitis B virus or human immunodeficiency virus infection, continued alcohol or drug abuse and those who also received immunosuppressive drug agents were excluded from the study. 348 patients fulfilled the above criteria and were included in the analysis. This study was approved by the ethics committee of the University Medical Center of Goettingen (initial approval number 4/8/93 and subsequent amendments). All patients gave their written informed consent to participate in the study in accordance with the ethical guidelines of the 1975 Declaration of Helsinki. Patients also gave their written informed consent to perform *EPO* rs1617640 and *ITPA* rs1127354 genetic testing. Further disease chronicity was defined histopathologically by using established criteria [20]. In patients, who refused liver biopsy, chronicity was documented by longitudinal observation and/or the results of clinical, biochemical and imaging results. Before the initiation of therapy, a liver biopsy was obtained from 249 patients. On the basis of histological, biochemical and imaging results 48 individuals had evidence of severe fibrosis and cirrhosis. 15 out of 99 individuals who refused liver biopsy had indirect signs of cirrhosis by clinical, biochemical and imaging results.

### Treatment regimen and definition of efficacy

Patients received 1 of 3 treatment regimens (Table 1): (1), PEG-IFN-α_2b_ 1.5 μg/kg/week (wk) (standard dose) or (2), PEG-IFN-α_2b_ 1.0 μg/kg/wk (low dose), both in combination with oral RBV dosed by body weight (40 – 65 kg, 800 mg/day; >65– 85 kg, 1000 mg/day; >85– 105 kg, 1200 mg/day; >105–125 kg, 1400 mg/day); or (3), PEG-IFN-α_2a_ 180 μg/wk plus oral RBV 1000 – 1200 mg/day dosed by body weight (<75 kg, 1000 mg/ day; ≥75 kg, 1200 mg/day). RBV dose was adjusted to body weight but not to viral genotypes, according to two recent studies in the field [21, 22]. PEG-IFN-α_2a_ and PEG-IFN-α_2b_ dose was reduced when WBC and/or platelet counts fell below 1,500×10^3^ cells/μl or 50,000×10^3^ cells/μl respectively. Dose modifications of weekly PEG-IFN-α_2a_ were made by decremental adjustments of 180 μg to 135 μg and 90 μg. PEG-IFN-α_2b_ dose was reduced to 1.0 μg/kg/week or replaced by 0.5 μg/kg/week PEG-IFN-α_2b_. RBV dose was reduced if Hb was <10 g/dl or when patients complained of symptoms. Dose modification of daily RBV dose was performed in decrements of 200 mg.

**Table 1:** **Baseline patient characteristics (n = 348)**

Female sex, n (%)	126 (36)
Age [median (IQR)] years	50 (43 – 58)
Ethnicity Caucasian, n (%)	346 (99)
HCV genotype 1/2/3 (%)	240/25/83 (69/7/24)
ALT [median (IQR)] U/l	49 (28 – 86)
Hb [median (IQR)] g/dl	15.1 (14.2 – 16)
Creatinine [median (IQR)] mg/dl	0.8 (0.7 – 0.9)
EPO^#^ [median (IQR)] mIU/ml*	7.8 (6 – 10.5)
Hepatitis activity mild, n (%)	170 (68)
Fibrosis absent or mild, n (%)	201 (81)
Severe Fibrosis or Cirrhosis^1^, n (%)	63^1^ (18)
Steatosis absent or mild, n (%)	224 (90)
Initial daily RBV dose^2^, n (%)	
800 mg	37 (10)
1.000 mg	125 (36)
1.200 mg	110 (32)
1.400 mg	76 (21)
PEG-IFN-α treatment, n (%)	
PEG-IFN-α_2a_	238 (68)
PEG-IFN-α_2b_ 1.0 μg/kg	30 (9)
PEG-IFN-α_2b_ 1.5 μg/kg	80 (23)
SVR by genotype 1/2/3, n (%)	101/20/68 (42/80/82)
*EPO* rs1617640 TT/TG/GG, n (%)	113/168/67 (33/48/19)
*ITPA* rs1127354 CC/CA/AA, n (%)	280/66/2 (80/19/1)

Data are given as median and interquartile range, if not indicated otherwise. 249 patients undergone histological evaluation; ^#^Pretreatment serum EPO measurement was available in 181 patients: *Normal range: 3.3-16.6 mIU/ml; ^1^48 patients with histological signs of severe fibrosis or cirrhosis and 15 patients with clinical, biochemical and imaging evidence of severe fibrosis or cirrhosis. ^2^Initial daily RBV dose was weight-based on a sliding scale in subjects’ baseline weight. *Abbreviations:* *HCV* hepatitis C virus, *γ-GT* gamma-glutamyltransferase, *ALT* alanine transaminase, *RBV* ribavirin, *PEG-IFN-α* pegylated interferon-α, *SVR* sustained virological response, *EPO* erythropoietin, *ITPA* inosine triphosphatase.

### Data collection and treatment of anemia

Clinical examination, total blood cell counts and routine biochemical tests and efficacy assessments were performed during the treatment period every 2 weeks during the first 12 weeks, then four-weekly until week 48 and, finally, at weeks 4 and 24 during follow-up. At these time-points, serum samples were obtained and stored at −20°C until further use. Serum samples were collected from 2003 to 2012.

When and how to treat anemia was essentially left at the discretion of the physician who treated the patient. Center specific standard operating procedures advised that treatment should be initiated when Hb dropped below 10 g/dl or when the patient complained of symptoms. At the discretion of the physician anemia could be treated by blood transfusions, epoetin-α supplementation, RBV dose reduction or a combination thereof. During treatment the physician in charge was unaware to patients’ *ITPA* and *EPO* genotypes and EPO serum concentrations as these analyses were performed only after completion of treatment.

### Study end points

In accordance with previous analyses [14] we analyzed Hb reduction of >3 g/dl. Differently to these previous analyses we did not only consider Hb reduction at week 4 weeks but also at week 12 as a composite endpoint, i.e., occurrence of an end point at either one of the time points. Furthermore, we analyzed RBV dose reduction, administration of blood transfusions or epoetin-α supplementation within 12 weeks of treatment as a composite event.

### Specific laboratory procedures

Detection of serum HCV-specific RNA by RT-PCR and determination of HCV genotypes were performed as described earlier [23, 24]. Serum HCV-RNA was monitored monthly.

Isolation of genomic DNA and single nucleotide polymorphism (SNP) genotyping were performed as described earlier [25]. Genotyping of *EPO* rs1617640 was performed by using the following primer: 36 μmol/l of each primer in each case; forward, 5′-AGC TAA GGT TTT ATG GCT TCT GGA A-3′; reverse 5′-GGT CTC CTG CTC TGG GAA TC-3′. Allelic discrimination was achieved by adding 8 μmol/l differentially fluorescent dye-labeled allele-specific minor groove binder probes (EPO: VIC, 5′-CTG AGC CAG AGG AGT GA-3′; FAM, 5′-CTG AGC CAG ATG AGT GA-3′). Genotyping of *ITPA* rs1127354 (ABI; NO: C_27465000) was performed according to the manufacturer’s instruction.

Serum levels of EPO were measured using the Quantikine human EPO enzyme linked immunosorbent assay (ELISA; R&D Systems, Articel-Nr: DEPOO). The assays were performed according to the manufacturer’s instructions.

### Statistical analyses

For this exploratory statistical analysis P-values of less than 0.05 were considered as statistically meaningful. In general, continuous variables are presented with median and interquartile range (IQR) and were analyzed by the non-parametric Mann–Whitney-U test [26]. Binary and categorical variables were compared by Pearsons chi-squared test or the Cochran-Armitage Trend Test in case of ordered categorical variables [27]. Hardy-Weinberg-equilibrium was tested by likelihood ratio test [28].

Multivariate logistic regression included variables that have been reported to influence Hb in patients on treatment, such as age, sex, and pre-treatment Hb, viral genotype, and ribavirin starting dose [29, 30]. The major allele of a SNP was considered to be the baseline allele (CC for *ITPA* rs1127354 and TT for *EPO* rs1617640). We primarily considered an additive genotype model for *ITPA* rs1127354 and a recessive model for *EPO* rs1617640 (with respect to the minor allele) as suggested from previous analyses [13, 14, 31], but dominant and co-dominant models were also considered. Backward stepwise model selection was performed based on the Akaike information criterion (AIC) [32]. The estimates are reported as odds ratios (OR) with confidence intervals and P-values based on the likelihood ratio. An OR above one indicates a higher risk of anemia in patients with the corresponding trait.

A linear model for the development of erythropoietin levels over time was fitted with an interaction factor of time and the minor allele of *EPO* rs1617640. A significant interaction of time and the *EPO* gene variant in this model indicates a different development of erythropoietin for patients being homozygous for the minor allele compared to patients being heterozygous or homozygous for the major allele. Because of repeated measurements we confirmed the analysis in a mixed model. P values cited were obtained from likelihood ratio test.

All statistical analyses were performed using the R language and environment for statistical computing version 2.15.2 [33].

## Results

### Patient characteristics

A total of 348 patients were included in this study. Baseline demographic, biochemical, and virological characteristics of the study cohort are listed in Table 1.

The two polymorphisms of interest, *EPO* rs1617640 and *ITPA* rs1127354, were genotyped in all patients (Table 1); genotype distributions met Hardy-Weinberg-equilibrium (*EPO* rs1617640 p = 0.75; *ITPA* rs1127354 p = 0.33). The resulting minor allele frequencies (MAF) of 0.434 and 0.101 for *EPO* rs1617640 (allele G) and *ITPA* rs1127354 (allele A), respectively, were close to those reported for healthy Caucasian controls [13, 14].

### Serum EPO concentrations and incidence of marked Hb decline with regard to *EPO*rs1617640 genotypes

**Figure 1:**
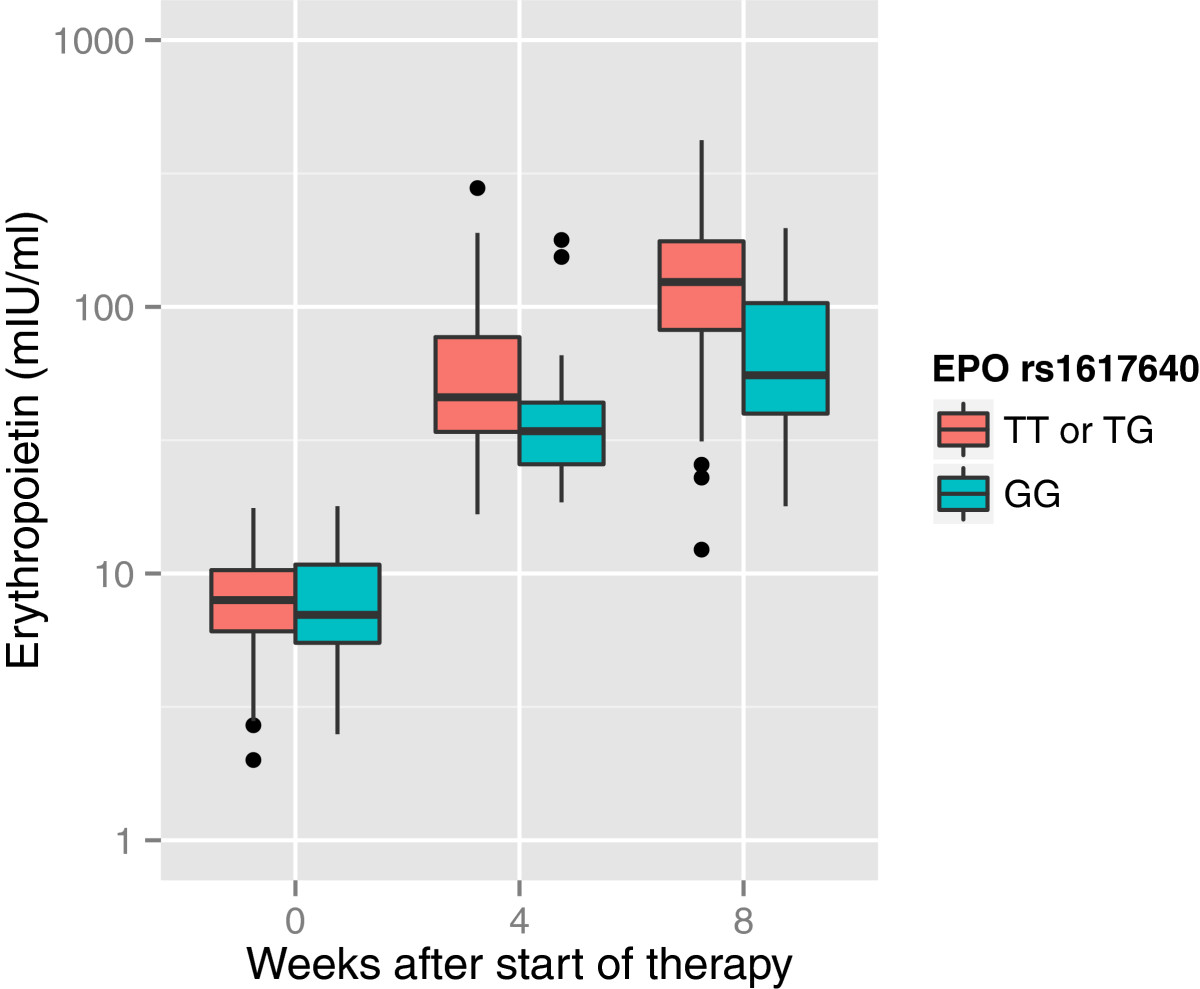
**Pre- and on-treatment serum EPO levels with regard to** ***EPO*** **rs1617640 genotypes.** Pretreatment serum EPO concentrations were equally distributed among major T allele carriers and minor G homozygotes. On-treatment serum EPO concentrations of *EPO* rs1617640 G homozygotes and T allele carriers at week 4 and 8 of therapy, however, differed significantly in a linear model. Data on serum EPO concentrations were available in 181, 112 and 78 individuals at week 0, 4 and 8, respectively. Medians and IQRs are given.

Serum EPO concentrations at baseline were available for 181 individuals, all of them found to be within the normal range (Table 1). During therapy, concentrations raised 5-fold by week 4 (median 43.2 mIU/ml, IQR 28.70 to 68.25) and 14-fold by week 8 (median 106.20 mIU/ml, IQR 65.45 to 160.5). *EPO* rs1617640 G homozygotes had similar baseline serum EPO concentrations when compared to T allele carriers (Figure 1). A linear model, however, revealed a lower rise over time in G homozygotes (p < 0.001 for interaction of time and gene variant in simple linear model and p = 0.008 in a linear mixed effects model, Figure 1).

**Figure 2:**
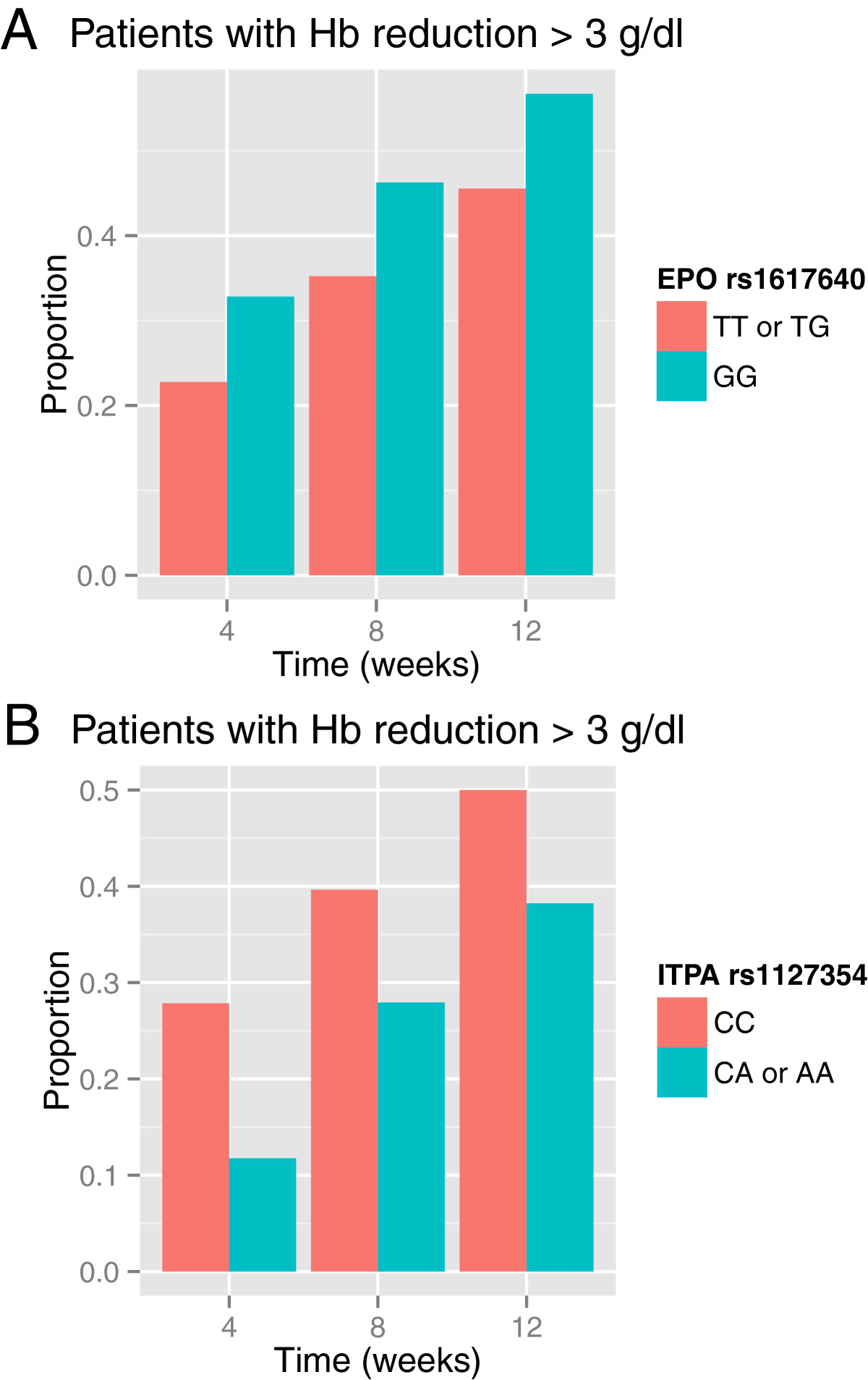
**Cumulative proportion of patients with an Hb reduction >3 g/dl during antiviral combination therapy with regard to** ***EPO*** **rs1617640 (A) and** ***ITPA*** **rs1127354 (B) genotypes.** A marked Hb reduction was more frequent among *EPO* rs1617640 minor allele G homozygotes and *ITPA* rs1127354 major allele C homozygotes than among *EPO* rs1617640 T allele and *ITPA* rs1127354 A allele carriers.

**Figure 3:**
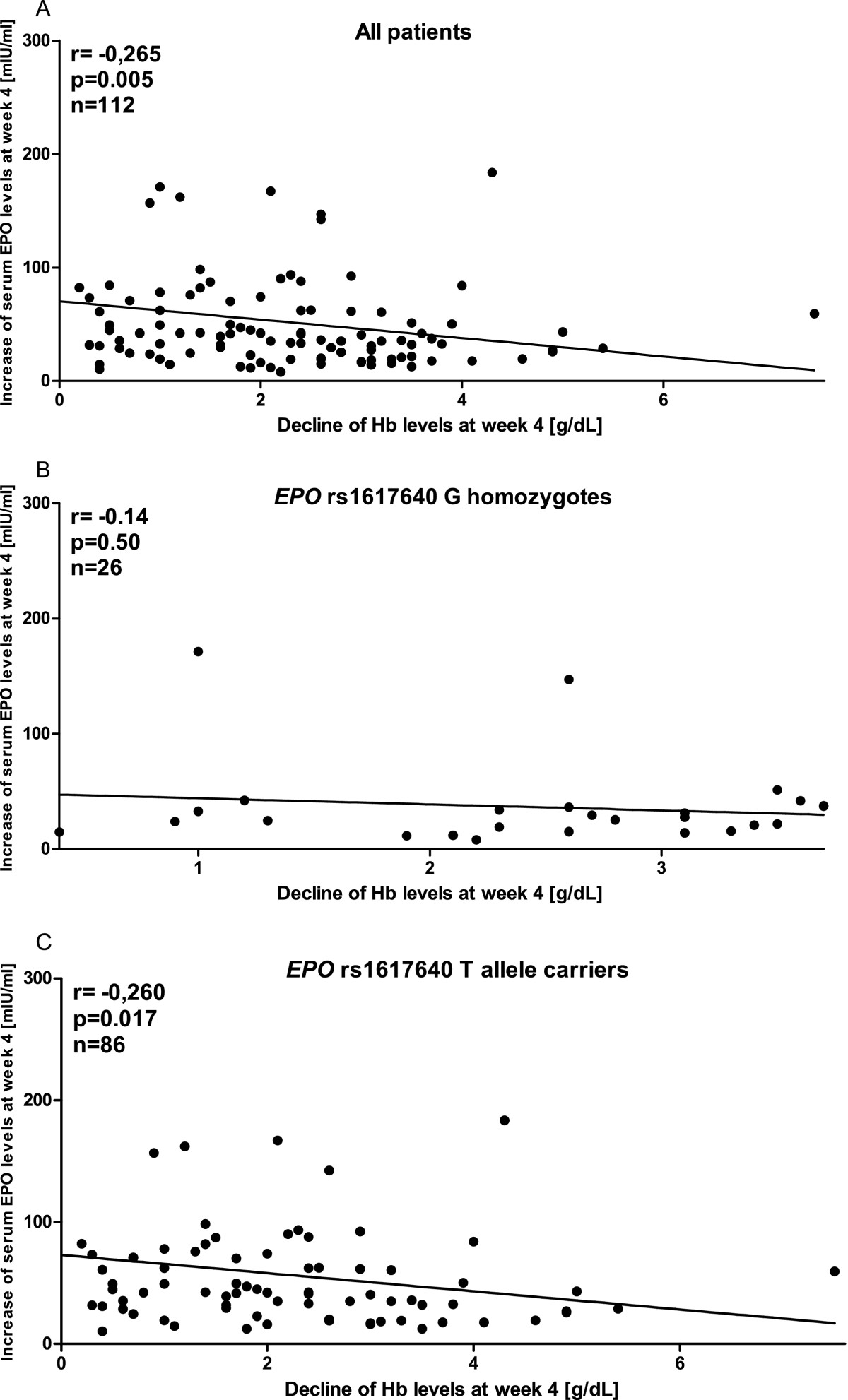
**Linear regression analysis of serum EPO levels and Hb levels at week 4.** This analysis revealed an inverse relationship between the increase of serum EPO levels and the decline of Hb levels in 112 patients of whom data on serum EPO levels were available **(A)**. This relationship is valid for carriers of the T allele **(C)** but not for G homozygous patients **(B)**. Correlation coefficients and levels of significance are given.

At baseline, patients’ median Hb concentration was within the normal range (15.1 g/dl, IQR 14.2 to 16.0 g/dl). Median Hb concentration declined at week 4, 8 and 12 by −1.8 (IQR 12.1 to 14.2 g/dl, p < 0.001), −2.4 (IQR 11.4 to 13.6 g/dl, p < 0.001) and −2.6 (IQR 11.1 to 13.4 g/dl, p < 0.001), respectively. The cumulative frequency of patients with Hb reductions > 3 g/dl at week 4, 8 and 12 was 25%, 32% and 40%, respectively (data not shown). Median baseline Hb levels of G homozygotes were 14.7 g/dl (IQR 14 to 15.6) and of T homo- and heterozygotes 15.2 (IQR 14.2 to 16.1) (p = 0.088). Median baseline hematocrit levels of G homozygotes were 44% (IQR 42 to 45) and of T homo- and heterozygotes 43% (IQR 41 to 46) (p = 0.48). With regard to *EPO* rs1617640 genotypes, G homozygotes experienced more frequently a marked Hb decline than T allele carriers (Figure 2A). In a univariate analysis, this difference did not reach statistical significance (p = 0.09, p = 0.09 and p = 0.1 for weeks 4, 8 and 12, respectively). In multivariate logistic regression analyses, *EPO* rs1617640 allele G associates with an increased risk of Hb reduction of more than 3 g/dl at week 4 (odds ratio (OR) 2.17, confidence interval (CI) 1.09 to 4.3, p = 0.025) (Table 2) and week 12 (OR 1.97, CI 1.07 to 3.66, p = 0.029) (Table 2) of therapy, respectively. A linear regression analysis revealed the increase of serum EPO levels to be inversely associated with the decline of Hb levels at week 4 (Figure 3A). Stratification for *EPO* rs1617650 genotypes revealed this inverse correlation to be valid for T allele carriers (Figure 3C) but not for G homozygotes (Figure 3B). Data thus support an impact of this polymorphism on the relationship of serum EPO and Hb levels. In addition, older age, higher Hb values and higher RBV dose at the onset of therapy significantly increase the risk of patients to have Hb reduction at 4 and 12 weeks (Table 2), whereas viral genotype had no significant effect on Hb reduction (data not shown).

**Table 2:** **Variables associated with Hb reduction > 3 g/dl**

	Univariate analysis		Multivariate analysis	
Characteristics	Odds ratio [95% CI]	*P*value	Odds ratio [95% CI]	*P*value
**Week 4**				
*EPO* rs1617640 (GG vs. TT/TG)	1.66 [0.92 – 2.94]	0.088	2.17 [1.09 – 4.30]	0.025
*ITPA* rs1127354 (additive)	0.35 [0.15 – 0.71]	0.007	0.32 [0.13 – 0.70]	0.007
Age (years)	0.97 [0.95 – 0.99]	0.0085	0.97 [0.95 – 1.00]	0.02
Sex (female vs. male)	0.75 [0.44 – 1.26]	0.285	1.64 [0.88 – 3.07]	0.12
Baseline Hb (g/dl)	2.24 [1.76 – 2.92]	< 0.001	2.50 [1.91 – 3.34]	< 0.001
RBV starting dose*	2.49 [1.03 – 7.34]	0.011	2.50 [1.03 – 7.34]	0.036
**Week 12**				
*EPO* rs1617640 (GG vs. TT/TG)	1.57 [0.92 – 2.70]	0.064	1.97 [1.07 – 3.66]	0.029
*ITPA* rs1127354 (additive)	0.60 [0.35 – 1.01]	0.058	0.58 [0.32 – 1.03]	0.067
Age (years)	0.97 [0.95 – 0.99]	0.002	0.97 [0.95 – 0.99]	0.004
Sex (female vs. male)	0.86 [0.55 – 1.33]	0.49	1.75 [1.04 – 2.99]	0.084
Baseline Hb (g/dl)	1.90 [1.55 – 2.35]	< 0.001	2.08 [1.67 – 2.64]	< 0.001
RBV starting dose*	2.09 [1.20 – 3.79]	0.011	2.18 [1.17 – 4.20]	0.016

*Abbreviations:* *EPO* erythropoietin, *ITPA* inosine triphosphatase, *RBV* ribavirin, *CI* confidence interval. *RBV starting dose is coded as an ordered categorical variable with levels of 800, 1000, 1200, and 1400 mg with linear increments.

### Clinical endpoints with regard to *EPO*rs1617640 genotypes

Epoetin-α supplementation, RBV dose reduction or blood transfusions were indicated in 14%, 5%, and 4% of patients, respectively. All three Hb reconstitution measures were analyzed as a composite event. An analysis with regard to *EPO* rs1617640 genotypes revealed 40% of G homozygotes to be affected by at least one of these events compared to only 14% of the T allele carriers (p < 0.001, Table 3). Also in multivariate logistic regression, the *EPO* rs1617640 G allele strongly associated with a higher risk of an event (p < 0.001) such as RBV dose reduction and epoetin-α supplementation. When we decompose the composite event and look at the single end-points we observed a significant effect of *EPO* rs1617640 on epoetin-α supplementation and RBV dose reduction (p < 0.001 for both in Pearsons chi-squared test), but not for blood transfusions (p = 0.366). Hb levels of *EPO* rs1617640 G homozygotes and the need for epoietin-α supplementation remained stable between week 4 (11 g/dl), 8 (11.4 g/dl) and 12 (11.5 g/dl), respectively. Other factors that are associated with the risk of a clinical event are sex (female sex: OR 0.41, CI 0.21 to 0.80, p = 0.003) and RBV starting dose (OR 1.18, CI 1.01 to 1.39, p = 0.036) but not baseline Hb (Table 4).

**Table 3:** **Cumulative proportion of Hb reconstitution measures with regard to EPO and ITPA genotypes**

EPO rs1617640	GG (n = 27)	40%	P < 0.001
	GT/TT (n = 39)	14%	
ITPA rs1127354	CC (n = 58)	21%	P = 0.079
	CA/AA (n = 8)	12%	

*Abbreviations:* *Hb* hemoglobin, *EPO* erythropoietin, *ITPA* inosine triphosphatase.

**Table 4:** **Variables associated with the combined clinical endpoint of RBV dose reduction, transfusion of erythrocyte concentrates, or administration of epoetin-α**

	Univariate analysis		Multivariate analysis	
Characteristics	Odds ratio [95% CI]	*P*value	Odds ratio [95% CI]	*P*value
*EPO* rs1617640 (GG vs. TT/TG)	4.19 [2.31 – 7.59]	< 0.001	4.14 [2.20 – 7.82]	< 0.001
Sex (female vs. male)	0.45 [0.24 – 0.83]	0.013	0.41 [0.21 – 0.80]	0.003
Baseline Hb (g/dl)	0.87 [0.70 – 1.09]	0.24	0.83 [0.65 – 1.07]	0.168
RBV start dose*	1.21 [1.04 – 1.40]	0.012	1.18 [1.01 – 1.39]	0.036

*Abbreviations:* *EPO* erythropoietin, *ITPA* inosine triphosphatase, *RBV* ribavirin, *CI* confidence interval. *RBV starting dose is coded as a continuous variable with an intercept at 800 and an increase of 1 in the model corresponding to 100 mg increase of the actual dose.

While our data revealed an association of *EPO* rs1617640 genotypes and the need for Hb reconstitution measures as one clinical endpoint, they did not unveil any relationship to baseline Hb level or to other clinical endpoints as histological stage of liver disease or antiviral treatment outcome (data not shown).

### Laboratory and clinical parameters with regard to *ITPA*rs1617640 variants

The overall incidence of Hb reduction of more than 3 g/dl increased steadily over a period of 12 weeks during treatment (Figure 2). *ITPA* rs1127354 C homozygotes showed an Hb reduction >3 g/dl at week 4, 8 and 12 of 27%, 39% and 50%, respectively (Figure 2B). The risk of decreasing Hb levels >3 g/dl was significantly higher in *ITPA* rs1127354 C homozygotes compared to T allele carriers during treatment at week 4 (p = 0.005), but less pronounced later at week 8 or 12 (p = 0.07 and 0.08).

The Cochran-Armitage trend test indicated an effect of *ITPA* rs1127354 C allele carriers on Hb reduction at week 4 and only marginally at week 12, with the minor allele A ameliorating anemia (p = 0.005 and p = 0.056, respectively). In multivariate logistic regression *ITPA* rs1127354 gene variant is associated with decreased risk of Hb reduction at week 4 (OR 0.32, CI 0.13 to 0.7, p = 0.007) but not at week 12 (OR 0.58, CI 0.32 to 1.03, p = 0.067) (Table 2). *ITPA* gene variation had no significant effect on clinical endpoints such as epoetin-α supplementation, RBV dose reduction or blood transfusions (p = 0.079) (Table 3). No interactions between *EPO* rs1617640 and *ITPA* rs1127354 could be shown by likelihood ratio test.

## Discussion

The major findings of the present study are: (1) serum EPO levels of all individuals increased significantly 5-fold at week 4 and 14-fold at week 8 compared to baseline, (2) *EPO* rs1617640 G homozygotes showed significantly lower serum EPO levels during antiviral treatment compared to T allele carriers, (3) besides age, baseline Hb levels and RBV dose, *EPO* rs1617640 G allele is independently associated with Hb decline during antiviral treatment, (4) in *EPO* rs1617640 G homozygotes the need of RBV dose reduction as well as epoetin-α supplementation was signi ficantly higher compared to T allele carriers, (5) *ITPA* rs1127354 gene variant rather associated with Hb reduction at week 4 but not at week 12 and did not increase the risk of epoetin-α supplementation, RBV dose reduction or blood transfusion.

Hb decline during antiviral treatment is a frequent side effect and the reason for it is probably multifactorial. IFN-α induces a significant and rapid dose-dependent Hb decline in CHC patients probably by causing an inhibition of hematopoietic stem cell proliferation [6, 34, 35]. Accumulation of RBV in red blood cells may aggravate anemia by inducing hemolysis.

The most important mediator of erythropoiesis is EPO. Several reports have examined serum EPO levels during antiviral treatment and could show that serum EPO levels are increasing up to 4-fold at week 4 in patients treated with PEG-IFN-α and RBV while Hb levels are declining [10, 12]. Our present study is consistent with these results in this respect. Here, we examined for the first time a single nucleotide polymorphism (SNP) within the *EPO* gene promoter, rs1617640 [13], in chronic hepatitis C patients who were undergoing antiviral treatment. The T allele of this polymorphism had been shown to be associated with higher levels of EPO in the vitreous body fluid of non-diabetic patients than the G allele [13]. The present study found *EPO* rs1617640 G homozygotes to have an attenuated serum EPO response compared to T allele carriers. Moreover, *EPO* rs1617640 G homozygotes also had higher incidence of significant Hb reduction at week 4 and 12. Finally, *EPO* rs1617640 G homozygotes had a significantly higher need of RBV dose reduction or epoetin-α supplementation, but not blood transfusion. The reason for this might be the relatively small sample number of patients who achieved blood transfusion.

Although this study investigated the *EPO* rs1617640 SNP with regard to a common side effect such as Hb decline of antiviral therapy in CHC patients, our findings might not be specific for therapy of CHC with RBV. This SNP might directly be involved in the regulation of the EPO response to acute Hb decline in other conditions as well. Here, the role of RBV might just be in inducing an “erythropoietic stress test” taking advantage of “controlled conditions” which are not typically achievable in human research. Therefore, further research (basic and clinical) should investigate the role of the *EPO* gene variation in various anemic diseases.

Interestingly, the Hb levels of *EPO* rs1617640 G homozygotes who were treated with epoetin-α remained stable between weeks 4 to 12. This observation suggests that substitution of EPO in patients whose *EPO* gene activation appears to be less stimulable than in others for genetic reasons might be a rational measure and thus superior to RBV dose reduction.

A genome-wide association study has described genetic variants that are associated with a decrease of Hb during antiviral combination therapy at week 4 [14]. This genome-wide association study (GWAS), however, did not report associations between Hb reduction and any SNP within the EPO gene. Indeed, the *EPO* rs1617640 was not present on the Illumina Human610-quad BeadChips. The only SNP in relative high linkage (r^2^: 0.865) with the *EPO* rs1617640 was rs221795 at a 34,037 basepair distance (http://www.broadinstitute.org/mpg/snap/). Moreover, GWASs are necessarily broad in scope. They search the entire genome for associations rather than focusing on small candidate areas and they do not necessarily identify all relevant SNPs [36]. Furthermore, all GWAS that evaluated SNPs for Hb decline while on treatment for CHC focused on end points at week 4 and could therefore only evaluate gene variations for short term Hb decline but not mid- to long-term Hb decline. This analysis shows that particularly short term Hb decline associates with *ITPA* gene variant while longer-term Hb decline appears to relate on *EPO* gene variant. This hypothesizes that short and long-term Hb decline on treatment for HCV may have somewhat different mechanisms.

The effect of baseline Hb on the reduction of Hb on treatment is explainable by the hypothesis that reduction of Hb due to RBV is relative and not absolute. Therefore higher baseline Hb is associated with higher incidence of Hb reduction of more than 3 g/dl, because a reduction of 3 g/dl is equivalent to 19% reduction when the baseline Hb is 16 g/dl but 23% when the baseline Hb is 13 g/dl. On the other side when the end-point is formulated as Hb reduction below 10 g/dl a higher baseline Hb is associated with a lower risk to reach this endpoint (for the same reasons). Furthermore, age is also a well-known risk factor for Hb reduction during antiviral therapy [37, 38].

The obvious constraint of this analysis is that this is a retrospective and explorative analysis of a registry data and not a formal trial. For this purpose *EPO* rs1617640 polymorphism should be evaluated in a prospective trial. Nevertheless, the consistency of these multiple analyses and results, i.e. association of *EPO* gene variant with lower serum erythropoietin increase, a higher risk of Hb reduction, and higher incidence of adverse events suggests that EPO may indeed play an hitherto unheralded role in the treatment of CHC.

In terms of new therapeutic options, especially in light of IFN-free regimens, 5% to 9% of patients who were treated with a DAA- and RBV-containing regimen showed increased Hb decline (<10 g/dl) compared to those who were not treated with a RBV-containing regimen [39–43]. Therefore, also for IFN-free regimens with RBV, EPO rs1617640 genotyping might be worth to be valuated for estimating a risk for a marked Hb decline. A need to terminate these new and costly treatment options because of serious Hb declines is critical not least in view of inhibitor-resistance mutations.

## Conclusion

*EPO* promoter rs1617640 genotypes, serum EPO concentration and *ITPA* rs1127354 genotypes might be promising parameters to be further evaluated in view of a risk assessment for Hb decline and the individuals’ capacity for an EPO response in IFN-α- and RBV-based therapy regimes.

## Authors’ original submitted files for images

Below are the links to the authors’ original submitted files for images. Authors’ original file for figure 1 Authors’ original file for figure 2 Authors’ original file for figure 3

## References

[1] McHutchison JG, Lawitz EJ, Shiffman ML, Muir AJ, Galler GW, McCone J, Nyberg LM, Lee WM, Ghalib RH, Schiff ER, Galati JS, Bacon BR, Davis MN, Mukhopadhyay P, Koury K, Noviello S, Pedicone LD, Brass CA, Albrecht JK, Sulkowski MS, IDEAL Study Team (2009). Peginterferon alfa-2b or alfa-2a with ribavirin for treatment of hepatitis C infection. N Engl J Med.

[2] Manns MP, McHutchison JG, Gordon SC, Rustgi VK, Shiffman M, Reindollar R, Goodman ZD, Koury K, Ling M, Albrecht JK (2001). Peginterferon alfa-2b plus ribavirin compared with interferon alfa-2b plus ribavirin for initial treatment of chronic hepatitis C: a randomised trial. Lancet.

[3] Fried MW, Shiffman ML, Reddy KR, Smith C, Marinos G, Goncales FL, Gonçales FL, Häussinger D, Diago M, Carosi G, Dhumeaux D, Craxi A, Lin A, Hoffman J, Yu J (2002). Peginterferon alfa-2a plus ribavirin for chronic hepatitis C virus infection. N Engl J Med.

[4] McHutchison JG, Manns MP, Muir AJ, Terrault NA, Jacobson IM, Afdhal NH, Heathcote EJ, Zeuzem S, Reesink HW, Garg J, Bsharat M, George S, Kauffman RS, Adda N, Di Bisceglie AM, PROVE3 Study Team (2010). Telaprevir for previously treated chronic HCV infection. N Engl J Med.

[5] Bacon BR, Gordon SC, Lawitz E, Marcellin P, Vierling JM, Zeuzem S, Poordad F, Goodman ZD, Sings HL, Boparai N, Burroughs M, Brass CA, Albrecht JK, Esteban R, HCV RESPOND-2 Investigators (2011). Boceprevir for previously treated chronic HCV genotype 1 infection. N Engl J Med.

[6] Peck-Radosavljevic M, Wichlas M, Homoncik-Kraml M, Kreil A, Hofer H, Jessner W, Gangl A, Ferenci P, Gangl A, Ferenci P (2002). Rapid suppression of hematopoiesis by standard or pegylated interferon-alpha. Gastroenterology.

[7] De Franceschi L, Fattovich G, Turrini F, Ayi K, Brugnara C, Manzato F, Noventa F, Stanzial AM, Solero P, Corrocher R (2000). Hemolytic anemia induced by ribavirin therapy in patients with chronic hepatitis C virus infection: role of membrane oxidative damage. Hepatology.

[8] Van Soest H, Renooij W, Van Erpecum KJ (2009). Clinical and basal aspects of anemia during antiviral therapy for hepatitis C. Ann Hepatol.

[9] Van Vlerken LG, Van SH, Janssen MP, Boland GJ, Drenth JP, Burger DM, Siersema PD, Van Erpecum KJ (2010). Suboptimal endogenous erythropoietin response in chronic hepatitis C patients during ribavirin and PEG interferon treatment. Eur J Gastroenterol Hepatol.

[10] Trivedi HS, Trivedi M (2004). Subnormal rise of erythropoietin in patients receiving interferon and ribavirin combination therapy for hepatitis C. J Clin Gastroenterol.

[11] Schmid M, Kreil A, Jessner W, Homoncik M, Datz C, Gangl A, Ferenci P, Peck-Radosavljevic M, Ferenci P, Peck-Radosavljevic M (2005). Suppression of haematopoiesis during therapy of chronic hepatitis C with different interferon alpha mono and combination therapy regimens. Gut.

[12] Durante ME, Marrone A, Saviano D, Del VC, Utili R, Ruggiero G (2003). Normal erythropoietin response in chronic hepatitis C patients with ribavirin-induced anaemia. Antivir Ther.

[13] Tong Z, Yang Z, Patel S, Chen H, Gibbs D, Yang X, Hau VS, Kaminoh Y, Harmon J, Pearson E, Buehler J, Chen Y, Yu B, Tinkham NH, Zabriskie NA, Zeng J, Luo L, Sun JK, Prakash M, Hamam RN, Tonna S, Constantine R, Ronquillo CC, Sadda S, Avery RL, Brand JM, London N, Anduze AL, King GL, Bernstein PS (2008). Promoter polymorphism of the erythropoietin gene in severe diabetic eye and kidney complications. Proc Natl Acad Sci U S A.

[14] Fellay J, Thompson AJ, Ge D, Gumbs CE, Urban TJ, Shianna KV, Little LD, Qiu P, Bertelsen AH, Watson M, Warner A, Muir AJ, Brass C, Albrecht J, Sulkowski M, McHutchison JG, Goldstein DB (2010). ITPA gene variants protect against anaemia in patients treated for chronic hepatitis C. Nature.

[15] Rembeck K, Waldenstrom J, Hellstrand K, Nilsson S, Nystrom K, Martner A, Lindh M, Norkrans G, Westin J, Pedersen C, Färkkilä M, Langeland N, Buhl MR, Mørch K, Christensen PB, Lagging M (2014). Variants of the inosine triphosphate pyrophosphatase gene are associated with reduced relapse risk following treatment for HCV genotype 2/3. Hepatology.

[16] Holmes JA, Roberts SK, Ali RJ, Dore GJ, Sievert W, McCaughan GW, Crawford DH, Cheng WS, Weltman MD, Bonanzinga S, Visvanathan K, Sundararajan V, Desmond PV, Bowden DS, Matthews GV, Thompson AJ, Crawford DH, Cheng WS, Weltman MD, Bonanzinga S, Visvanathan K, Sundararajan V, Desmond PV, Bowden DS, Matthews GV, Thompson AJ, CHARIOT Study Group (2014). ITPA Genotype Protects Against Anemia During Peginterferon And Ribavirin Therapy But Does Not Influence Virological Response. Hepatology.

[17] Boglione L, De NA, Cusato J, Cariti G, Di PG, D’Avolio A (2014). Significant early higher ribavirin plasma concentrations in patients receiving a triple therapy with pegylated interferon, ribavirin and telaprevir. J Viral Hepat.

[18] Ahmed WH, Furusyo N, Zaky S, Eldin AS, Aboalam H, Ogawa E, Murata M, Hayashi J (2013). Pre-treatment role of inosine triphosphate pyrophosphatase polymorphism for predicting anemia in Egyptian hepatitis C virus patients. World J Gastroenterol.

[19] Ogawa E, Furusyo N, Nakamuta M, Kajiwara E, Nomura H, Dohmen K, Takahashi K, Satoh T, Azuma K, Kawano A, Tanabe Y, Kotoh K, Shimoda S, Hayashi J, Kyushu University Liver Disease Study (KULDS) Group (2013). Clinical milestones for the prediction of severe anemia by chronic hepatitis C patients receiving telaprevir-based triple therapy. J Hepatol.

[20] Mihm S, Fayyazi A, Hartmann H, Ramadori G (1997). Analysis of histopathological manifestations of chronic hepatitis C virus infection with respect to virus genotype. Hepatology.

[21] Jacobson IM, Brown RS, Freilich B, Afdhal N, Kwo PY, Santoro J, Becker S, Wakil AE, Pound D, Godofsky E, Strauss R, Bernstein D, Flamm S, Pauly MP, Mukhopadhyay P, Griffel LH, Brass CA, Becker S, Wakil AE, Pound D, Godofsky E, Strauss R, Bernstein D, Flamm S, Pauly MP, Mukhopadhyay P, Griffel LH, Brass CA, WIN-R Study Group (2007). Peginterferon alfa-2b and weight-based or flat-dose ribavirin in chronic hepatitis C patients: a randomized trial. Hepatology.

[22] Ascione A, De Luca M, Tartaglione MT, Lampasi F, Di Costanzo GG, Lanza AG, Picciotto FP, Marino-Marsilia G, Fontanella L, Leandro G (2010). Peginterferon alfa-2a plus ribavirin is more effective than peginterferon alfa-2b plus ribavirin for treating chronic hepatitis C virus infection. Gastroenterology.

[23] Mihm S, Hartmann H, Fayyazi A, Ramadori G (1996). Preferential virological response to interferon-alpha 2a in patients with chronic hepatitis C infected by virus genotype 3a and exhibiting a low gamma-GT/ALT ratio. Dig Dis Sci.

[24] Amanzada A, Goralczyk A, Moriconi F, Blaschke M, Schaefer IM, van TD, Thiel D, Mihm S, Ramadori G (2011). Ultra-rapid virological response, young age, low gamma-GT/ALT-ratio, and absence of steatosis identify a subgroup of HCV Genotype 3 patients who achieve SVR with IFN-alpha(2a) monotherapy. Dig Dis Sci.

[25] Amanzada A, Schneider S, Moriconi F, Lindhorst A, Suermann T, van Thiel DH, Mihm S, Ramadori G (2012). Early anemia and rapid virological response improve the predictive efficiency of IL28B-genotype for treatment outcome to antiviral combination therapy in patients infected with chronic HCV genotype 1. J Med Virol.

[26] Hollander M, Pena EA (2004). Nonparametric Methods in Reliability. Stat Sci.

[27] Ryu E, Agresti A (2008). Modeling and inference for an ordinal effect size measure. Stat Med.

[28] Evett IW, Lambert JA, Buckleton JS, Weir BS (1996). Statistical analysis of a large file of data from STR profiles of British Caucasians to support forensic casework. Int J Legal Med.

[29] Ochi H, Maekawa T, Abe H, Hayashida Y, Nakano R, Kubo M, Tsunoda T, Hayes CN, Kumada H, Nakamura Y, Chayama K (2010). ITPA polymorphism affects ribavirin-induced anemia and outcomes of therapy–a genome-wide study of Japanese HCV virus patients. Gastroenterology.

[30] Thompson AJ, Fellay J, Patel K, Tillmann HL, Naggie S, Ge D, Urban TJ, Shianna KV, Muir AJ, Fried MW, Afdhal NH, Goldstein DB, McHutchison JG (2010). Variants in the ITPA gene protect against ribavirin-induced hemolytic anemia and decrease the need for ribavirin dose reduction. Gastroenterology.

[31] Thompson AJ, Santoro R, Piazzolla V, Clark PJ, Naggie S, Tillmann HL, Patel K, Muir AJ, Shianna KV, Mottola L, Petruzzellis D, Romano M, Sogari F, Facciorusso D, Goldstein DB, McHutchison JG, Mangia A (2011). Inosine triphosphatase genetic variants are protective against anemia during antiviral therapy for HCV2/3 but do not decrease dose reductions of RBV or increase SVR. Hepatology.

[32] Venables JP (2002). Alternative splicing in the testes. Curr Opin Genet Dev.

[33] Song X, Zhang BL, Liu HM, Yu BY, Gao XM, Kang LY (2011). IQMNMR: Open source software using time-domain NMR data for automated identification and quantification of metabolites in batches. BMC Bioinformatics.

[34] Wang Q, Miyakawa Y, Fox N, Kaushansky K (2000). Interferon-alpha directly represses megakaryopoiesis by inhibiting thrombopoietin-induced signaling through induction of SOCS-1. Blood.

[35] Lengfelder E, Berger U, Hehlmann R (2000). Interferon alpha in the treatment of polycythemia vera. Ann Hematol.

[36] Couzin-Frankel J (2010). Major heart disease genes prove elusive. Science.

[37] Reau N, Hadziyannis SJ, Messinger D, Fried MW, Jensen DM (2008). Early predictors of anemia in patients with hepatitis C genotype 1 treated with peginterferon alfa-2a (40KD) plus ribavirin. Am J Gastroenterol.

[38] Sulkowski MS, Wasserman R, Brooks L, Ball L, Gish R (2004). Changes in haemoglobin during interferon alpha-2b plus ribavirin combination therapy for chronic hepatitis C virus infection. J Viral Hepat.

[39] Wyles DL, Rodriguez-Torres M, Lawitz E, Shiffman ML, Pol S, Herring RW, Massetto B, Kanwar B, Trenkle JD, Pang PS, Zhu Y, Mo H, Brainard DM, Subramanian GM, McHutchison JG, Habersetzer F, Sulkowski MS, Massetto B, Kanwar B, Trenkle JD, Pang PS, Zhu Y, Mo H, Brainard DM, Subramanian GM, McHutchison JG, Habersetzer F, Sulkowski MS (2014). All-oral combination of ledipasvir, vedroprevir, tegobuvir, and ribavirin in treatment-naive patients with genotype 1 HCV infection. Hepatology.

[40] Kowdley KV, Gordon SC, Reddy KR, Rossaro L, Bernstein DE, Lawitz E, Shiffman ML, Schiff E, Ghalib R, Ryan M, Rustgi V, Chojkier M, Herring R, Di Bisceglie AM, Pockros PJ, Subramanian GM, An D, Svarovskaia E, Hyland RH, Pang PS, Symonds WT, McHutchison JG, Muir AJ, Pound D, Fried MW, ION-3 Investigators (2014). Ledipasvir and sofosbuvir for 8 or 12 weeks for chronic HCV without cirrhosis. N Engl J Med.

[41] Afdhal N, Zeuzem S, Kwo P, Chojkier M, Gitlin N, Puoti M, Romero-Gomez M, Zarski JP, Agarwal K, Buggisch P, Foster GR, Bräu N, Buti M, Jacobson IM, Subramanian GM, Ding X, Mo H, Yang JC, Pang PS, Symonds WT, McHutchison JG, Muir AJ, Mangia A, Marcellin P, ION-1 Investigators (2014). Ledipasvir and sofosbuvir for untreated HCV genotype 1 infection. N Engl J Med.

[42] Afdhal N, Reddy KR, Nelson DR, Lawitz E, Gordon SC, Schiff E, Nahass R, Ghalib R, Gitlin N, Herring R, Lalezari J, Younes ZH, Pockros PJ, Di Bisceglie AM, Arora S, Subramanian GM, Zhu Y, Dvory-Sobol H, Yang JC, Pang PS, Symonds WT, McHutchison JG, Muir AJ, Sulkowski M, Kwo P, ION-2 Investigators (2014). Ledipasvir and sofosbuvir for previously treated HCV genotype 1 infection. N Engl J Med.

[43] Zeuzem S, Dusheiko GM, Salupere R, Mangia A, Flisiak R, Hyland RH, Illeperuma A, Svarovskaia E, Brainard DM, Symonds WT, Subramanian GM, McHutchison JG, Weiland O, Reesink HW, Ferenci P, Hézode C, Esteban R, VALENCE Investigators (2014). Sofosbuvir and ribavirin in HCV genotypes 2 and 3. N Engl J Med.

[CR44] The pre-publication history for this paper can be accessed here:http://www.biomedcentral.com/1471-2334/14/503/prepub

