# Supplementary figures and images for: Erythropoietin rs1617640 G allele associates with an attenuated rise of serum erythropoietin and a marked decline of hemoglobin in hepatitis C patients undergoing antiviral therapy
Source: BMC Infect Dis. 2014 Sep 17;14:503. doi: 10.1186/1471-2334-14-503 (PMC4175618; doi:10.1186/1471-2334-14-503)

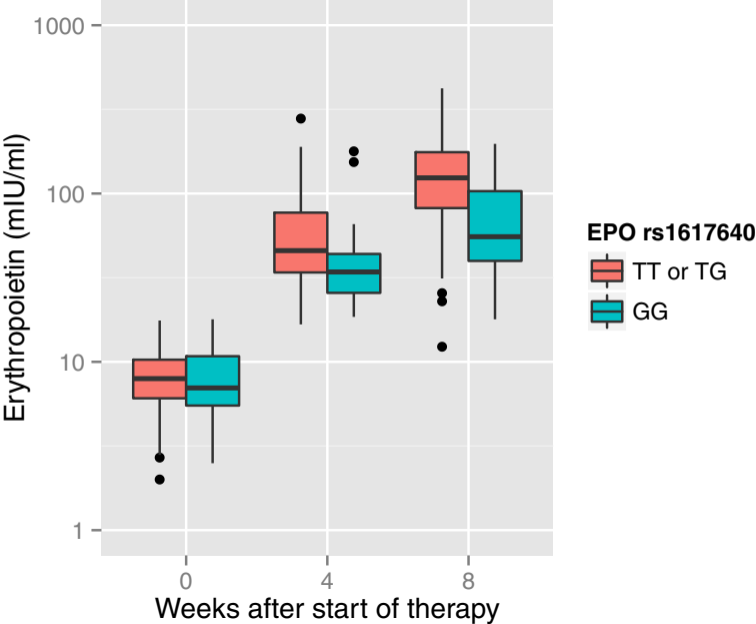

Supplement: Supplementary file 1 — Authors’ original file for figure 1 [file 12879_2014_3805_MOESM1_ESM.pdf]

# A Patients with Hb reduction > 3 g/dl

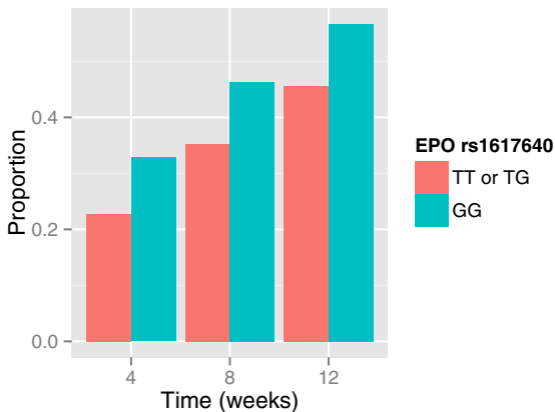

# B Patients with Hb reduction > 3 g/dl

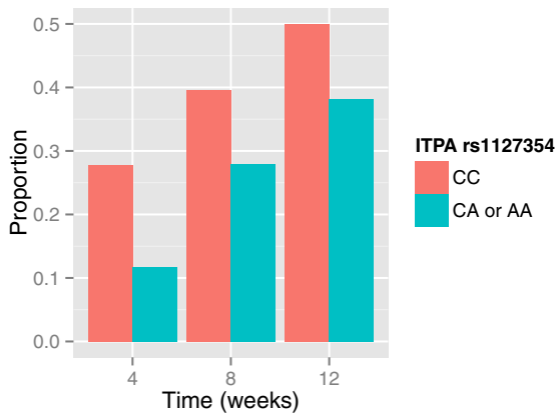

Supplement: Supplementary file 2 — Authors’ original file for figure 2 [file 12879_2014_3805_MOESM2_ESM.pdf]

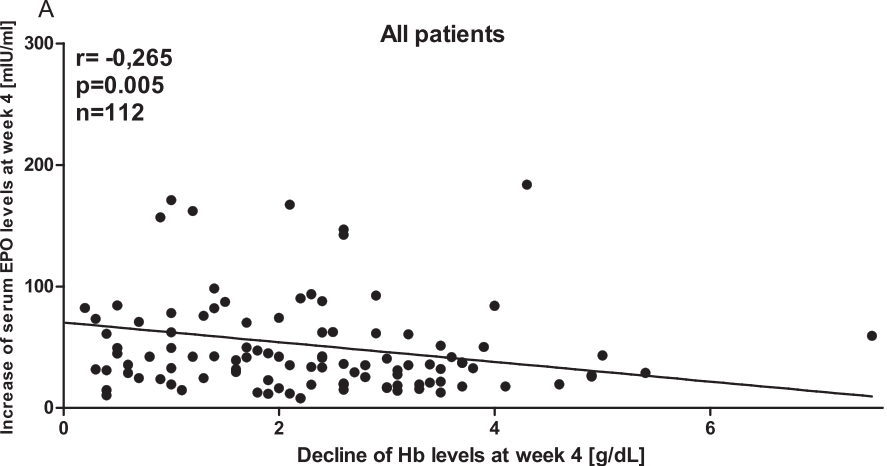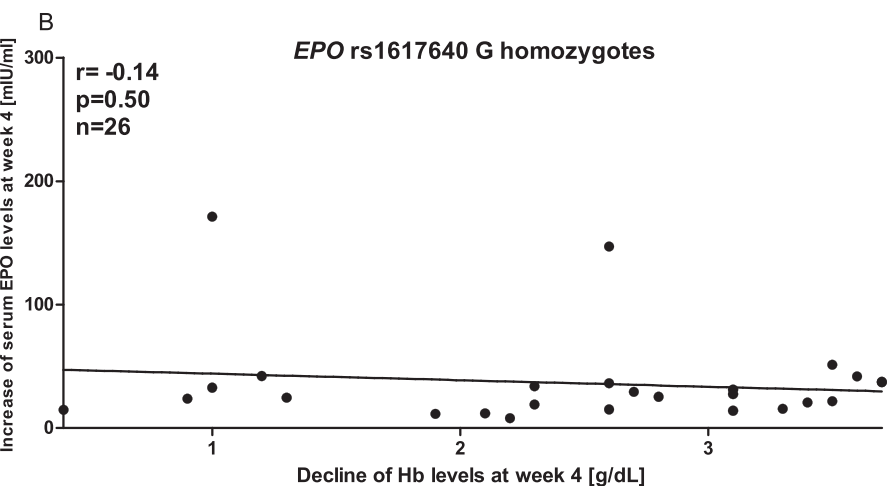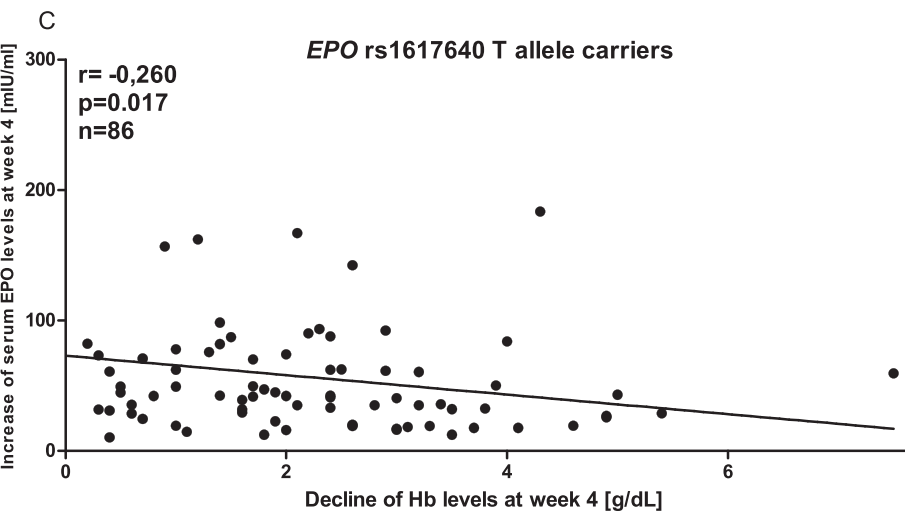

Supplement: Supplementary file 3 — Authors’ original file for figure 3 [file 12879_2014_3805_MOESM3_ESM.pdf]
